# Temperamental Dimensions in Early Childhood: Gender Differences and Their Relationship to Emotional and Behavioral Disorders in a Longitudinal Study

**DOI:** 10.3390/children12070946

**Published:** 2025-07-18

**Authors:** Teresa Navarro-Ariza, Lidia Infante-Cañete, Dolores Madrid-Vivar, Agustín Wallace Ruiz, Elena Alarcón-Orozco

**Affiliations:** 1 Department of Evolutive and Educational Psychology, Universidad de Málaga, 29071 Málaga, Spain; marnavari@uma.es (T.N.-A.); lidiainfante@uma.es (L.I.-C.); 2 Department of Didactics and School Organization, Universidad de Málaga, 29071 Málaga, Spain; lmadrid@uma.es; 3 Department of Psychobiology and Methodology of Behavioral Sciences, Universidad de Málaga, 29071 Málaga, Spain; awallace@uma.es

**Keywords:** temperament, gender, internalizing, externalizing, stability, change

## Abstract

**Background**: This longitudinal study aims to explore the stability and changes in child temperament dimensions between the ages of three and six, analyzing their relationship with emotional and behavioral problems, differentiated by gender. **Method**: This study involved 24 boys and 25 girls from various early childhood education centers in Málaga, Spain. To assess temperament, the Spanish adaptation of the Children’s Behavior Questionnaire was used, while emotional and behavioral problems were evaluated using the SPECI Screening for Emotional and Behavioral Problems in Children. **Results**: Findings indicate that 9 out of the 15 temperament dimensions remained stable, while 4—anger/frustration, attentional focusing, perceptual sensitivity, and sadness—showed significant changes in the total sample. The gender-specific analysis revealed different developmental patterns for boys and girls. Girls exhibited changes in attentional focusing, fear, and perceptual sensitivity, while boys showed changes in anger/frustration and attentional focusing. In addition, girls scored higher in discomfort and fear, whereas boys stood out in activity levels. Regarding behavioral problems, girls’ externalizing symptoms were significantly associated with attentional focusing and smiling/laughter, while internalizing symptoms were linked to low-intensity pleasure and perceptual sensitivity. **Conclusions**: These findings highlight the importance of addressing temperament from a gender-differentiated perspective when designing educational and family interventions aimed at promoting socioemotional development.

## 1. Introduction

Child temperament has been widely studied in developmental psychology due to its fundamental role in shaping the personality and socioemotional adjustment throughout life [1]. Temperament influences multiple areas of child development, including emotional regulation and susceptibility to emotional and behavioral issues [2]. It has also been related to areas such as eating [3], sleep [4], or the risk of developing psychopathological symptoms from an early age [5].

### 1.1. Definition and Dimensions of Temperament

Temperament is defined as a set of basic dispositions that manifest in the domains of activity, affectivity, attention, and self-regulation. These dispositions emerge early in life and result from complex interactions between genetic, biological, and environmental factors [6]. Moreover, temperament is considered an early form of the individual’s socioemotional behavior [7], including patterns of cognitive organization and even motor behavior [8].

### 1.2. Rothbart’s Classification

On this basis, Ref. [9] proposed a classification of temperament into three factors: positive affectivity, negative affectivity, and effortful control. Positive affectivity includes emotions such as joy, enthusiasm, and activity levels, which tend to promote social interaction and the exploration of the environment [10]. Negative affectivity encompasses emotions such as fear, sadness, anger, irritability or discomfort, and difficulties in returning to a calm state, which may predispose individuals to withdrawal or confrontational behaviors in stressful situations [11]. Finally, effortful control refers to the ability to inhibit impulsive responses and sustain attention on specific tasks, including the capacity to shift attention, perceptual sensitivity, the expression of low-intensity pleasure, and inhibitory control—skills that are essential for self-regulation and social adjustment [11,12] (see Table 1).

These traits represent relatively stable dispositions or tendencies in the way children respond emotionally, interact with their environment, and regulate their behavior. They reflect basic patterns of emotional and behavioral responses that emerge early in life and significantly influence social interactions and individuals’ adaptive capacity [10]. In fact, despite the changes inherent to development and interactions with the environment, numerous studies have emphasized the stability of temperamental traits over the lifespan [13] and thus can help predict the social and emotional adjustment in later stages [9], as well as even predict personality traits two decades later [14].

During the first months of life, temperamental traits tend to show relative stability up to approximately 18 months of age [15]. Dimensions such as positive anticipation, the activity level, impulsivity, and shyness also tend to remain stable until shortly before the age of 3. However, traits related to self-regulation and negative affectivity begin to decline from that age onwards [16].

Furthermore, Ref. [9] found that the activity level in one-year-old infants is positively associated with impulsivity and motor activity at age seven, suggesting a continuity in these traits. Similarly, children who exhibit a rapid approach to strangers or novel situations in early childhood tend to experience greater difficulties with inhibitory and attentional control at later stages. These findings reinforce the idea that, while some temperamental dimensions are relatively stable, others may evolve depending on developmental processes and environmental interactions.

There is evidence for the stability of temperament dimensions that relate to positive affectivity, such as approachability, sociability, and high activity levels [11,17,18] in infancy and early childhood. This refers to children who are typically cheerful, active, and seek the company of others, including unfamiliar people [19]. A higher tendency to approach strangers or new situations during the first year of life predicts greater difficulties with inhibitory control in the preschool stage. In this sense, Ref. [9] has conducted research with children aged three to six, linking these temperamental dimensions to tasks involving inhibition and attentional control. Ref. [20] also found associations between the dimensions of anger and inhibitory control and attention tasks in 4-year-old children.

### 1.3. Relationship Between Temperamental Dimensions and Behavior

Temperamental dimensions are associated with various behavioral patterns. In this sense, extraversion may be linked to emotional problems and aggression during childhood [21], whereas high levels of activity and emotionality predict risk-taking behaviors in adolescence [22]. Children with high levels of frustration and impulsivity may experience an increase in these temperamental traits when exposed to negative parenting and educational practices [23]. Similarly, high levels of behavioral control are positively associated with good social competence and more context-appropriate responses, whereas low levels of control are related to behavioral problems with peers or authority figures.

Although certain temperamental dimensions show stability, temperament also exhibits flexibility, allowing adaptations to the environment and responses to its demands [24]. Thus, studying both the stability and flexibility of temperament is crucial for identifying and predicting traits that may predispose individuals to emotional and behavioral problems [9]. Likewise, personal experience and maturation are factors that influence the development of temperament, while temperament itself shapes how the environment interacts with the individual. In this regard, the maternal perception of temperament has been shown to influence communicative interactions [25], dyadic engagement [26], and even the provision of developmental opportunities [27], with primary caregivers tending to respond more positively when children have an easier temperament [28]. Other factors such as the parenting practices, family structure, or social class have also been shown to be related to temperamental patterns [29]. More specifically, children from lower socioeconomic backgrounds tend to express greater negative affectivity in their temperament, characterized by fear, anger, sadness, and calm regulation [30]. These studies suggest that temperament may function more as a moderating variable than as a primary construct, operating in a bidirectional relationship with interactive factors such as family educational models.

### 1.4. Gender Differences in Temperamental Dimensions: Changes in Attentional Focusing, Fear, Perceptual Sensitivity, and Emotional Regulation

Numerous studies have identified significant differences between boys and girls in relation to temperamental dimensions. Girls appear to exhibit more fear than boys and, on the other hand, lower activity levels than boys [31]. Other research shows that girls have greater inhibitory control and attentional focusing than boys [32], while boys appear to be more vulnerable to externalizing behavioral problems [33]. According to [34], boys display more risk-taking and confrontational behaviors than girls, whereas girls tend to be rated by teachers as less aggressive toward peers and more cooperative [35]. Ref. [36] also concluded that girls have a greater competence in controlling and allocating attention. These differences with respect to gender are argued on the basis of biological characteristics [37], as well as cultural influences or differing parenting practices in boys than in girls [38].

### 1.5. Need for Longitudinal Studies

Despite the importance of studying temperament in childhood, most research on temperament has focused on cross-sectional samples [21,39]. While these methodologies are useful for identifying patterns at different stages, they limit our understanding of how these differences evolve throughout child development. In contrast, longitudinal studies offer a deeper perspective by allowing the same cohorts to be followed over time, as in the works of [40,41]. Such studies are crucial to determine whether certain temperamental characteristics are stable or evolve as a result of maturation or environmental factors.

The scarcity of recent longitudinal studies on temperament in early childhood can be attributed to the time, effort, and resources required to conduct such research, which represents a significant barrier for many researchers. Although studies have been conducted on temperament in infants [42,43] and adolescents [44,45], longitudinal research focusing on the 3 to 6 year age range remains limited, with notable exceptions including the work of [3,46].

Therefore, it is essential to address this research gap, as longitudinal studies not only allow us to identify patterns of stability and change in temperament but also provide a better understanding of temperament development and the maturational processes involved in it [43]. As suggested by [33], adopting a longitudinal perspective contributes to understanding how temperament interacts with the environment and how it influences social and emotional adjustments in later stages.

In summary, childhood represents a crucial stage in the formation of temperamental traits that will influence the socioemotional adjustment and behavior throughout development [47]. In addition, although gender differences in temperament have been documented, most studies have been cross-sectional and have not examined how these differences evolve with age or their impact on socioemotional adjustments [32]. It has been suggested that girls tend to show greater inhibitory control and attentional focusing, while boys exhibit higher levels of activity and impulsivity [48]. However, it remains unclear to what extent these differences persist or change with age.

Given this context, this study focuses on analyzing the stability and changes in child temperament dimensions between the ages of three and six, considering gender differences and their relationship with emotional and behavioral problems. In doing so, it seeks to identify which dimensions act as protective factors and which may represent risk factors, potentially leading to positive or negative outcomes in socioemotional adjustments. This understanding will enable more effective prevention strategies to be established.

### 1.6. Objectives and Hypotheses

The objective of this research is to explore the stability and changes in child temperament dimensions, taking into account gender differences and their relationship with socioemotional adjustments. To this end, the following specific objectives are established: (1) to identify which temperamental dimensions remain more stable at the age of 3 to 6 years; (2) to determine the stability/change in temperamental dimensions according to gender; and (3) to evaluate the influence of temperament dimensions on emotional and behavioral problems based on gender.

Based on these objectives, the following hypotheses are proposed:

**H1:**  *Significant stability is expected in most temperament dimensions between the ages of three and six.*

**H2:**  *A statistically significant gender difference is expected to be found with respect to the following temperamental dimensions: anger/frustration, attentional focusing, shyness, fear and perceptual sensitivity, with girls obtaining higher levels of attentional focusing, fear, and perceptual sensitivity.*

**H3:**  *Significant gender differences are expected in how temperament dimensions relate to emotional and behavioral problems, with internalizing symptoms being more prevalent in girls and externalizing symptoms in boys.*

## 2. Materials and Methods

### 2.1. Participants

The final study sample consisted of 49 children (24 boys and 25 girls) from three early childhood education centers in the province of Málaga, Spain. Participants were selected through purposive sampling, considering centers with similar sociodemographic characteristics.

Initially, the study had 75 participants, but due to the longitudinal nature of the design, 26 subjects dropped out of the study for reasons such as changing schools, relocating, or incomplete questionnaires. Sample attrition is a frequent limitation in longitudinal studies, as the follow-up of the same individuals over an extended period may be affected by various factors.

The inclusion criterion was to have completed the two evaluation phases. Exclusion criteria included the presence of some type of neurodevelopmental diagnosis, being under 3 years of age at the initial evaluation, and failure to complete the evaluation scales.

### 2.2. Instruments

To assess temperament, we used the Children’s Behavior Questionnaire (CBQ) [49], specifically the Spanish-adapted version developed by the University of Murcia (for the purposes of this paper, we will adhere to the terminology of the original English version). This questionnaire has demonstrated high internal consistency in previous studies, with Cronbach’s alpha values ranging from 0.72 to 0.89 in Spanish samples. This questionnaire gathers information provided by parents to evaluate temperament in children aged 3 to 7 years. It consists of 91 items rated on a 7-point Likert scale and is grouped into 15 temperament dimensions, as shown in Table 1.

For emotional and behavioral problems, the Screening for Emotional and Behavioral Problems in Children (SPECI, by its Spanish acronym) was used. It has shown reliability coefficients ranging from 0.85 to 0.91, confirming its validity for evaluating emotional problems in childhood [50]. It is completed by teaching staff, who are able to identify the presence or warning signs of emotional and behavioral problems in children aged 5 to 12. It consists of 10 items describing behaviors associated with withdrawal, somatization, anxiety, dependent-child pattern, thought problems, attention–hyperactivity, disruptive behavior, academic performance, depression, and violent behavior. Items are rated on a Likert scale from 0 to 2 points, based on the intensity with which the referenced behavior occurs (none, somewhat, very much).

The scale assesses internalizing and/or externalizing behaviors and problems. Internalizing problems are composed of items related to symptoms or behaviors that reflect difficulties or maladaptive ways of dealing with emotional conflicts internally. These include issues such as thought problems, withdrawal, depression, dependent-child behaviors, somatic complaints, or anxiety responses. On the other hand, externalizing symptoms refer to emotional conflicts expressed outwardly, such as disruptive and violent behaviors, inattention, hyperactivity symptoms, and academic performance issues.

### 2.3. Design

A longitudinal single-group repeated-measures design was used.

### 2.4. Procedure

This study began with initial meetings with the management teams of the different centers to present the objectives of the intervention and propose the data collection process. After the proposal was accepted, it was forwarded to the early childhood education team and subsequently approved by the School Board. Informational meetings were then held with the students’ families, during which the purpose of the study was explained. In these meetings, the signed consent of the parents or legal guardians of the minors was requested.

Data collection was carried out through the school tutors. The questionnaires were administered individually to preserve privacy and allow both anonymity and free responses from the participants. Data were collected longitudinally during the early childhood education stage (3 to 6 years of age). Temperament questionnaires were administered to parents at the beginning and end of the educational stage—that is, when the children were three and six years old, respectively. The SPECI questionnaire, addressed to school tutors, was administered in the last year (6 years) to each of the schoolchildren.

### 2.5. Data Analysis

In order to further examine the stability and change in the variables studied, descriptive analyses based on measures of central tendency were conducted. The normality of the data was assessed using the Kolmogorov–Smirnov test. As the distribution met the criteria for normality, Pearson’s correlation coefficient was used to evaluate the stability of the scores across time. These analyses were complemented with mean comparison tests using Student’s *t*-test. To analyze the relationship between variables, the statistical software package SPSS version 26 was used.

## 3. Results

Table 2 shows the descriptive statistics of the overall sample and according to gender for each of the variables studied at the age of 3 and 6 years, respectively.

To address the first objective—identifying which temperament dimensions remain most stable between ages three and six in each participant—a correlation study was conducted. The results (see Table 3) show that 9 out of the 15 dimensions of child temperament exhibit a moderate correlation between the two measurements. These dimensions include the activity level, anger/frustration, discomfort, high-intensity pleasure, impulsivity, inhibitory control, low-intensity pleasure, perceptual sensitivity, and shyness.

To further examine the stability and change, a descriptive analysis was conducted using measures of the central tendency and mean comparison tests with the Student’s *t*-test (see Table 4) for the total sample. The results show significant differences in anger/frustration (AF), attentional focusing (AT), perceptual sensitivity (PS), and shyness (SH).

Thus, stability was found in 9 out of the 15 dimensions—however, significant changes were observed in 4 dimensions, indicating that the temperament stability is not uniform across all dimensions.

The second objective focuses on identifying the stability of temperament dimensions over time according to gender (see Table 4). To this end, an analysis of differences in temperament dimensions by gender between ages three and six was conducted using descriptive statistics (measures of central tendency) and mean comparison tests with the Student’s *t*-test. The results indicate significant differences in boys in the dimensions of anger/frustration (AF), attentional focusing (AT), and impulsivity (IM), with lower scores in AF, higher scores in AT, and lowers scores in IM. In relation to girls, significant differences were obtained in the following dimensions: attentional focusing (AT), fear (FE), perceptual sensitivity (PS), and shyness (SH), obtaining significantly higher mean scores in all dimensions except shyness (see Table 4).

In addition, a study has been conducted to determine how different boys and girls are at age 6. The results show that the main differences are in the3 activity level (AL2), discomfort (DI2), and fear (FE2) (see Table 5).

The third objective aims to assess whether certain temperament dimensions influence the socioemotional adjustment between the ages of three and six. To this end, a correlation analysis was conducted, which showed that there is no significant correlation between the SPECI scores and the CBQ dimensions when analyzing the full sample without distinguishing by gender (see Table 6), except for a correlation between the internalizing dimension and FE2. This relationship was also explored by gender. In the case of boys, the results indicate that there is no significant correlation between temperament dimensions and the internalizing and externalizing factors of the SPECI. However, in girls significant correlations were identified between the externalizing factor and the dimensions of attentional focusing (AF) and smiling/laughter (SL), as well as between the internalizing factor and the dimensions of low-intensity pleasure (LP) and perceptual sensitivity (PS).

To determine whether internalizing and externalizing factors vary by gender (see Table 7, Table 8 and Table 9), a mean comparison test was conducted using the Student’s *t*-test. The results revealed significant differences in both factors, with higher scores in boys. Additionally, the differential item analysis of the SPECI, using the Mann–Whitney U test, showed significant differences in items 3, 4, and 5, which correspond to the categories of anxiety, the dependent-child pattern, and thought problems, respectively, in which boys scored higher.

## 4. Discussion

The main objective of this study was to analyze the dimensions of child temperament during the developmental stage from ages three to six, assessing their stability and variations, as well as their relationship with emotional and behavioral problems, taking into account gender differences. A series of specific objectives were established to guide the analysis of the results.

Regarding the first objective—identifying which temperament dimensions remain most stable between the ages of three and six—the results (see Table 3) show that 9 out of the 15 dimensions of childhood temperament exhibit a moderate correlation. These dimensions include the activity level, anger/frustration, discomfort, high-intensity pleasure, impulsivity, inhibitory control, low-intensity pleasure, perceptual sensitivity, and shyness. These results are consistent with previous research [9,47,49], which also suggest that certain temperament traits are shown to be stable across development [13]. In this regard, there is evidence that dimensions such as the activity level, high-intensity pleasure, and impulsivity show a stability in early and middle childhood [17], while others, such as anger/frustration, discomfort, and fear, along with self-regulation decrease significantly from early life to the preschool years []. Therefore, the temperament stability is not uniform across all dimensions, suggesting that some traits may be more strongly influenced by biological factors or socialization patterns.

Regarding the second objective—determining the changes that occur between ages three and six—the results indicate significant changes in four dimensions: anger/frustration, attentional focusing, perceptual sensitivity, and sadness. These findings are consistent with the existing literature, which suggests that these dimensions change due to socioemotional development [49] and the maturation of executive functioning [51,52]. In addition, the family environment plays a key role in modulating these temperament dimensions [47]. Specifically, Refs. [32,43,47,49] suggest that changes in the anger/frustration dimension are associated with advances in executive functions, such as the acquisition of self-regulation strategies, the development of cognitive skills, and neuroplasticity processes that allow for the better control of emotional responses. Likewise, Ref. [47] emphasizes that a supportive family environment and appropriate parenting practices play an important role in shaping this dimension. However, other studies have shown that when children exhibit high levels of anger/frustration at an early age, this trait tends to persist over a longer period of time [53]. With regard to attentional focusing, a faster development has been observed in children who receive cognitive stimulation and emotional support, which aligns with previous research on the development of the executive attention network [53]. Furthermore, the literature suggests that this dimension tends to stabilize around age 4, when children begin engaging in more specific tasks that require sustained attentional control [54]. As for perceptual sensitivity and sadness, these dimensions are shaped by the development of emotional self-regulation and are influenced by environmental and family factors. Therefore, a positive environment fosters the development of prosocial behaviors and healthy emotional adjustment [11,55,56]. These findings highlight the role of environmental factors in socioemotional development and, consequently, in self-regulation, influencing how children perceive and manage stimuli [55,56].

Regarding the determination of stability and change in temperament dimensions from ages three to six by gender, the results indicate notable variations compared to the total sample when conducting a differential analysis. In boys, changes were observed in the dimensions of anger/frustration, attentional focusing, and impulsivity, while in girls, changes were found in attentional focusing, fear, perceptual sensitivity, and sadness. When considering the differences between boys and girls at age 6, the results show significant differences in activity levels, discomfort, and fear: boys tend to score higher in activity levels, while girls score higher in discomfort and fear. These findings are consistent with previous research, such as that of [32,47], which notes that boys generally have more difficulty controlling their impulses than girls, particularly in situations involving frustration or stimulation. Conversely, girls are more effective at inhibiting impulsive behavior and regulating their actions. Ref. [32] also points out that girls at an early age tend to demonstrate a greater attentional capacity and self-regulation than boys, including skills such as waiting, planning, and controlling impulses. These dimensions are associated with greater inhibitory control, which is linked to compliance with rules as well as the development of empathy, guilt, and shame later on [11]. On the other hand, these differences may also reflect social and cultural aspects. According to [2,47], certain forms of socialization promote more advanced self-regulation skills in girls from early childhood. The cultural environment and parenting practices affect these gender differences, as social expectations may reinforce the attentional capacity in girls, while boys are often socialized toward more active, physical play. This may influence their ability to sustain prolonged attention on specific tasks, as highlighted by [57]. Attentional focusing, which tends to be higher in girls, enables them to respond to stimuli in a more controlled way and to develop self-control skills that support adaptive behavior [58]. In relation to the dimension of impulsivity, Ref. [59] observed that these gender differences are more pronounced in childhood but tend to decrease during adolescence. Again, these results can be explained by a combination of biological and environmental factors.

The third objective—assessing whether certain temperament dimensions predict emotional and behavioral problems with internalizing or externalizing symptoms at age 6, considering gender as a moderating variable—yielded different results depending on whether the total sample or gender-specific groups were analyzed. Thus, in the total sample, no significant relationships were generally observed between temperament dimensions and emotional or behavioral problems. This result could be explained by the nonclinical nature of the sample, which aligns with results reported by [50]. However, when analyzing differences between boys and girls, distinct patterns emerged. In boys, no significant correlations were found between temperament dimensions and the internalizing and externalizing factors, although boys scored higher overall than girls on both factors—particularly in the categories of anxiety, the dependent-child pattern, and thought problems. These findings are consistent with those reported by [60], suggesting that boys may be more prone to exhibiting problematic behaviors, both internalizing and externalizing, as supported by research such as [61], which highlights the higher prevalence of behavioral and externalizing disorders in boys. In contrast, for girls, a moderate correlation was found between externalizing symptoms and the temperament dimensions of attentional focusing and smiling/laughter, as well as between internalizing symptoms and the dimensions of low-intensity pleasure and perceptual sensitivity. Previous studies have shown that girls tend to score higher in variables related to emotional awareness and other socioemotional traits, such as prosocial attitudes and empathy, which may support adaptation. On the other hand, girls also appear to express greater internalizing symptomatology, including depression and emotional distress [62]. These findings partially confirm hypothesis H3, as the expected relationship between temperament and internalizing symptoms was identified in girls, while no clear patterns were observed in boys. Previous research has documented that high perceptual sensitivity and elevated negative affectivity may be associated with a greater vulnerability to anxiety and depression [50].

These differences between boys and girls with respect to the expression of internalizing and externalizing problems may also be due to educational and parenting factors, as gender differences may be interpreted as social roles or expectations that affect behavior [60]. The sociocultural environment in which boys and girls grow up has its own characteristics and values, to which children must adapt [63].

In conclusion, this study reinforces the importance of child temperament as a key factor in socioemotional development during the ages of three to six. The findings demonstrate the stability of certain temperament dimensions in relation to gender, as well as their differential influence on emotional and behavioral problems in boys and girls. These differences not only reflect the influence of biological factors on the stability of temperament dimensions but also highlight the impact of parenting practices and the sociocultural environment on those dimensions that depend on the development of executive functions. The differences observed between boys and girls, and the specific dimensions in which these differences occur, reinforce the need to design targeted interventions tailored to temperamental and gender-specific characteristics, thus directly addressing our objectives of identifying stable and modifiable traits that predict socioemotional outcomes and informing educational and family practices to promote balanced development and minimize the occurrence of both internalizing and externalizing problems during this crucial stage of childhood development.

Moreover, this study identified specific temperament dimensions that function as protective factors. In particular, higher levels of effortful control—including attentional focusing and inhibitory control—as well as lower levels of negative affectivity dimensions, such as fear and sadness, were associated with better socioemotional adjustment, especially among girls.

This study presents several strengths that reinforce its validity and relevance. One of its main strengths lies in the longitudinal design and the differential analysis by gender. The longitudinal approach allowed the observation of the same individuals throughout their developmental period between the ages of three and six. This design provided a more comprehensive and dynamic perspective, facilitating the identification of temperament dimensions that remained stable or changed over time, while minimizing the selection bias and ensuring a greater consistency in the results. Moreover, this study highlighted how certain temperament dimensions, as well as their relationship with internalizing and externalizing problems, differ significantly when analyzed using the overall sample versus a gender-differentiated approach. This approach emphasizes the importance of studying temperament from a perspective that considers gender-specific traits, which is crucial for understanding socioemotional developmental trajectories.

On the other hand, this study also presents some limitations that should be acknowledged. First, the relatively small sample size may limit the generalizability of the findings. Future research could aim to replicate these results with larger and more representative samples.

Although the findings provide valuable insights, the representativeness of the sample is limited. The children included in this study were recruited from three early childhood education centers in Málaga with similar sociodemographic profiles. Therefore, caution is advised when generalizing the results to broader populations. Future studies should aim to replicate the findings with larger and more diverse samples and, where possible, compare outcomes with established normative data.

Another limitation concerns the exclusive use of caregiver reports to assess temperament and emotional/behavioral issues. While these reports are valuable, the inclusion of direct assessments or observational measures in future studies would provide a more objective perspective and help reduce potential response biases [64].

Secondly, although this study analyzed the temperament stability, it did not control environmental factors such as the parenting quality or socioeconomic status, which could influence the variability of temperamental traits.

Moreover, it would be relevant to extend the follow-up beyond age six in order to assess whether changes in temperament persist into middle childhood and adolescence.

### 4.1. Educational Implications

The identification and understanding of child temperament are essential for families, educators, and mental health professionals, as they allow for a more accurate approach to individual needs in early childhood and their projection into adolescence. This knowledge can support the implementation of effective strategies and tools to promote social and emotional development and well-being [8]. In addition to highlighting the stability and change in temperament dimensions, the results of this study point to differential development based on gender, emphasizing the importance of considering this variable when designing interventions aimed at improving emotionally appropriate social interactions [65]. For example, since girls tend to show greater perceptual sensitivity, teachers may foster emotional regulation strategies in the classroom, such as mindfulness techniques, to help them manage sensory overstimulation. On the other hand, boys’ tendency to express higher levels of anger/frustration suggests the relevance of positive discipline strategies and early childhood training in conflict resolution skills.

### 4.2. Future Research

Future research on child temperament should explore the interaction between biological and sociocultural factors, including how parenting practices and gender expectations influence temperament stability and change. Additionally, it is important to investigate which specific strategies may help prevent internalizing and externalizing problems.

## Figures and Tables

**Table 1:** Factors and dimensions of child temperament.

FACTORS	DIMENSIONS	COD	DESCRIPTION
POSITIVE AFFECTIVITY	Activity level	AL	Amount and intensity of motor activity, primarily gross motor activity.
	High-intensity pleasure	HP	High-intensity pleasure refers to the amount of enjoyment or pleasure experienced in situations involving stimuli characterized by intensity, speed, novelty, etc.
	Impulsivity	IM	Speed with which the child initiates a response to a stimulus.
	Smiling/laughter	SL	Amount of expression of positive affectivity in response to stimuli or changes in stimuli.
	Shyness (-)	SH	Inhibition and discomfort in situations of social approach or involving novelty for the child.
	Positive anticipation	PA	How the child responds when an enjoyable activity is anticipated.
NEGATIVE AFFECTIVITY	Soothability (-)	SO	Ability to calm down and recover after a situation of excitement and high intensity.
	Fear	FE	Amount of discomfort, worry, or nervousness that arises in anticipation of discomfort, pain, or other situations the child perceives as threatening (negative affectivity).
	Anger/frustration	AF	Behavior of the child in frustrating or angry situations (negative affectivity).
	Sadness	SA	Mood or low energy experienced by the child in response to suffering or disappointment associated with the feeling of loss of a desired object (negative affectivity).
	Discomfort	DI	Sensory characteristics present in the environment, such as sound, light intensity, movement, etc.
EFFORTFUL CONTROL	Low-intensity pleasure	LP	Amount of pleasure and enjoyment in situations where the stimulus is low in intensity, weak, slow, or incongruent.
	Perceptual sensitivity	PS	Ability to perceive stimuli from the environment despite their low intensity.
	Attentional focusing	AT	Ability to maintain full attention on task.
	Inhibitory control	IC	Ability to make a planned response or inhibit an approach response when instructed or when the situation is unfamiliar.

**Table 2:** Descriptive data according to gender.

Dimensions	Three-Year-Olds						Six-Year-Olds					
	Total Sample		Boys		Girls		Total Sample		Boys		Girls	
	M	DE	M	DE	M	DE	M	DE	M	DE	M	DE
AL	28.9	6.3	30.8	6.65	27.1	5.36	28.8	5.9	30.5	5.05	27.2	6.31
AF	25.9	5.8	26.8	6.87	25.0	4.60	24.3	6.4	24.4	7.37	24.2	5.41
PA	30.4	3.9	31.2	4.52	29.6	3.15	31.1	3.9	31.4	4.15	30.7	3.66
AF	24.9	7.0	24.0	7.40	25.8	6.63	27.8	5.4	27.3	5.14	28.3	5.63
DI	27.4	7.4	26.3	7.59	28.4	7.27	26.7	7.3	24.8	7.38	28.4	6.96
SO	27.6	6.3	28.5	6.09	26.8	6.46	27.7	6.4	27.0	7.40	28.3	5.30
FE	25.5	6.8	25.7	7.48	25.3	6.29	26.2	6.3	24.5	6.83	27.8	5.32
HP	26.0	4.9	26.9	4.87	25.2	4.91	25.3	6.3	26.3	6.89	24.3	5.55
IM	26.7	5.7	27.9	6.35	25.6	4.96	26.0	6.1	25.7	5.68	26.3	6.56
IC	26.7	5.5	25.5	5.57	27.9	5.31	27.8	5.5	27.0	5.37	28.6	5.64
LP	46.0	5.2	46.4	6.10	45.6	4.18	45.7	4.3	45.7	4.53	45.8	4.07
PS	33.6	5.1	33.5	5.49	33.7	4.88	35.0	4.0	34.2	4.37	35.7	3.53
SA	29.9	5.8	29.4	6.25	30.3	5.34	30.8	5.1	30.4	6.11	31.2	4.08
SH	23.8	5.9	23.5	5.42	24.2	6.44	21.6	7.9	21.6	8.36	21.6	7.66
SL	32.8	4.4	33.2	4.91	32.4	3.95	33.1	5.0	32.9	5.71	33.3	4.28

AL: Activity level; AF Anger/frustration; PA: Positive anticipation; AT: Attentional focusing; DI Discomfort; SO: Soothability; FE: Fear; HP: High-intensity pleasure; IM: Impulsivity; IC: Inhibitory control; LP: Low-intensity pleasure; PS: Perceptual sensitivity; SA: Sadness; SH: Shyness; and SL: Smiling/laughter.

**Table 3:** Correlation matrix of temperament dimensions.

	AL	AF	PA	AT	DI	SO	FE	HP	IM	IC	LP	PS	SA	SH	SL
AL2	0.624 ***														
AF2		0.621 ***													
PA2			0.352 *												
AT2				0.390 **											
DI2					0.466 ***										
SO2						0.279									
FE2							0.325 *								
HP2								0.491 ***							
IM2									0.496 ***						
IC2										0.577 ***					
LP2											0.634 ***				
PS2												0.585 ***			
SA2													0.195		
SH2														0.662 ***	
SL2															0.388 **

Nota * *p* ˂ 0.05, ** *p* ˂ 0.01, and *** *p* ˂ 0.001.

**Table 4:** Descriptive analysis of temperament dimensions at ages 3 and 6.

Dimension	Total Sample		Boys		Girls	
	*t*	*p*	*t*	*p*	*t*	*p*
AL-AL2	0.110	0.913	0.219	0.414	−0.159	0.437
AF-AF2	**2.081**	0.043	**2.052**	0.026	0.824	0.209
PA-PA2	−1.040	0.303	−0.243	0.405	−1.150	0.130
AT-AT2	**−2.910**	0.005	**−2.116**	0.022	**−1.965**	0.031
DI-DI2	0.696	0.490	0.861	0.199	0.030	0.488
SO-SO2	−0.012	0.990	1.115	0.138	−0.833	0.193
FE-FE2	−0.633	0.530	0.651	0.260	**−2.053**	0.025
HP-HP2	0.932	0.356	0.513	0.306	0.846	0.203
IM-IM2	0.822	0.415	**1.707**	0.050	−0.725	0.237
IC-IC2	−1.500	0.140	−1.533	0.069	−0.613	0.272
LP-LP2	0.457	0.649	0.828	0.208	−0.149	0.442
PS-PS2	**−2.225**	0.031	−0.797	0.217	**−2.409**	0.012
SA-SA2	−0.931	0.356	−0.593	0.279	−0.734	0.235
SH-SH2	**2.635**	0.011	1.374	0.091	**2.465**	0.010
SL-SL2	−0.393	0.695	0.295	0.385	−1.037	0.155

AL2; Activity level; AF2: Anger/frustration; PA2: Positive anticipation, AT2; Attentional focusing, DI2: Discomfort; SO2: Soothability; FE2: Fear; IM2: Impulsivity; IC2: Inhibitory control; HP2: High-intensity pleasure; LP2: Low-intensity pleasure; PS2: Perceptual sensitivity; SA2: Sadness; SH2: Shyness; SL2; and Smiling/laughter.

**Table 5:** Temperament dimensions over time by gender.

Dimension	Total Sample	
	*t*	*p*
AL2	2.016	0.025
AF2	0.111	0.456
PA2	0.619	0.269
AT2	−0.612	0.271
DI2	−1.742	0.044
SO2	−0.700	0.243
FE2	−1.877	0.034
HP2	1.088	0.141
IM2	−0.351	0.363
IC2	−0.962	0.120
LP2	−0.061	0.237
PS2	−1.302	0.995
SA2	−0.550	0.292
SH2	0.003	0.997
SL2	−0.309	0.758

* *p* < 0.05; AL2; Activity level; AF2: Anger/frustration; PA2: Positive anticipation, AT2; Attentional focusing, DI2: Discomfort; SO2: Soothability; FE2: Fear; IC2: Inhibitory control; LP2: Low-intensity pleasure; PS2: Perceptual sensitivity; SA2: Sadness; SH2: Shyness; SL2; and Smiling/laughter.

**Table 6:** Correlations between SPECI and CBQ dimensions in the total sample.

	AL2	AF2	PA2	AT2	DI2	SO2	FE2	HP2	IM2	IC2	PB2	SP2	SA2	SH2	SL2
**Externalizing**	0.140	0.182	0.118	−0.205	0.049	0.138	0.229	0.032	0.085	−0.097	0.166	0.093	0.213	0.056	−0.059
**Internalizing**	0.062	−0.139	−0.064	−0.014	0.69	0.063	−0.293 *	−0.034	−0.048	−0.050	0.009	0.049	0.056	0.022	0.164

* *p* < 0.05; ** *p* <0.01; and *** *p* < 0.001.

**Table 7:** Correlations between SPECI and CBQ dimensions in the boys’ sample.

	AL2	AF2	PA2	AT2	DI2	SO2	FE2	HP2	IM2	IC2	LP2	PS2	SA2	SH2	SL2
**Externalizing**	0.055	0.109	0.154	−0.048	0.151	0.191	0.357	0.015	0.239	0.046	0.231	0.325	0.304	−0.059	0.096
**Internalizing**	−0.039	−0.290	−0.148	0.263	0.299	0.144	−0.290	−0.032	0.026	0.152	0.225	0.391	0.199	0.082	0.342

* *p* < 0.05; ** *p* <0.01; and *** *p* < 0.001.

**Table 8:** Correlations between SPECI and CBQ dimensions in the girls’ sample.

	AL2	AF2	PA2	AT2	DI2	SO2	FE2	HP2	IM2	IC2	LP2	PS2	SA2	SH2	SL2
**Externalizing**	0.114	0.392	−0.011	−0.472 *	0.077	0.126	0.252	−0.067	−0.101	−0.276	0.074	−0.254	0.079	0.309	−0.421 *
**Internalizing**	−0.022	0.175	−0.005	−0.384	0.088	−0.005	−0.104	−0.216	−0.132	−0.275	−0.404 *	−0.481 *	−0.211	−0.090	−0.169

* *p* < 0.05; ** *p* <0.01; and *** *p* < 0.001.

**Table 9:** Dimensions and items of the SPECI according to gender.

	M	SD	BOYS	GIRLS	Statistical	*p*
Externalizing	3.61	1.18	3.94	3.29	2.04	0.047
Internalizing	6.56	1.68	7.01	6.11	1.96	0.056
SPECI	9.29	1.67				
SPECI 1	1.15	0.49	1.215	1.0769	276	0.213
SPECI 2	0.94	0.37	0.999	0.8838	292	0.349
SPECI 3	1.16	0.42	1.288	1.0385	239	0.024
SPECI 4	1.25	0.55	1.382	1.1154	231	0.024
SPECI 5	1.02	0.24	1.082	0.9615	275	0.045
SPECI 6	1.28	0.52	1.397	1.1658	251	0.102
SPECI 7	1.08	0.34	1.164	1.0041	274	0.129
SPECI 8	1.14	0.40	1.214	1.0769	266	0.121
SPECI 9	1.04	0.34	1.047	1.0385	302	0.493
SPECI 10	0.10	0.30	0.169	0.0426	271	0.111

## Data Availability

The data presented in this study are available on request from the corresponding author due to ethical and privacy restrictions.
